# Parametric Study on Scarf Patch Repairs for Shipboard Composite Structures

**DOI:** 10.3390/ma19081644

**Published:** 2026-04-20

**Authors:** Panpan Liang, Guanbo Wang, Qingchang Guo, Maojun Li, Pan Gong

**Affiliations:** 1 State Key Laboratory of Advanced Design and Manufacturing Technology for Vehicle, Hunan University, Changsha 410082, China; 2 State Key Laboratory of Tribology in Advanced Equipment, Tsinghua University, Beijing 100084, China; 3 State Key Laboratory of Materials Processing and Die & Mold Technology, School of Materials Science and Engineering, Huazhong University of Science and Technology, Wuhan 430074, China

**Keywords:** composites, repair method, cut-out depth, finite element analysis

## Abstract

This study focuses on the of key engineering parameters for the repair of shipboard carbon fiber reinforced polymer composite structures using a scarf patch repair configuration. A three-dimensional finite element model was developed to systematically analyze the effects of repair location (center-symmetric, diagonal-asymmetric, and edge-unidirectional) and cut-out depth (2.0 mm, 3.0 mm, and 4.0 mm) on the mechanical response of the repair structure. The results indicate that although the local stress level of the center-symmetric repair is slightly higher, it provides a continuous load transfer path with more balanced stress distribution, demonstrating the best overall mechanical performance. When the cut-out depth is 3.0 mm, the repair structure achieves an optimal balance between stress uniformity and displacement coordination, effectively reducing the risk of early adhesive layer failure and local buckling. This study identifies the optimal parameter combination for scarf patch repairs, providing important theoretical foundations and references for the design of repair processes and the standardization of engineering practices in shipboard composite structures.

## 1. Introduction

In the patch repair of shipboard carbon fiber reinforced polymer (CFRP) structures, the scarf repair, with its continuously varying geometric configuration, demonstrates superior overall mechanical performance compared to stepped repairs in terms of alleviating stress concentration, controlling overall deformation, and improving interface strain coordination. This provides a clear theoretical basis for selecting the repair form [1]. However, applying this theoretical foundation to engineering practice still faces key challenges [2]. The repair effectiveness is not only influenced by the macro geometry but also by the specific cut-out location and cut-out depth. The repair location (the relative position of the damaged area within the parent structure) and cut-out depth (the ratio of the damaged material removal depth to the parent laminate thickness) play a decisive role in determining the repair structure’s load-bearing capacity and processing costs. Therefore, it is crucial to systematically study the effects of repair location and cut-out depth on the repair performance based on the selection of the scarf repair as the advantageous configuration.

The repair location directly determines the path and efficiency of load transfer from the patch to the adhesive layer and then to the parent laminate. However, in real ship structures, the damage may occur at the center, near the edges, or in asymmetrical complex locations. In existing composite repair studies, whether adhesive or patch repairs are used, most research is based on idealized center-symmetric damage models. Hou et al. [3] evaluated the response behavior of different damage locations under low-velocity impact conditions, indicating that different repair locations affect energy absorption and load transfer differently. When the repair location is farther from the center of the laminate, peel stress increases, and energy absorption decreases with increasing distance from the center. Tie et al. [4] further pointed out that when the damage hole is 20 mm to the right of the center of the laminate, different shaped patches were used to repair the damage, and the results show that the shape of the patch repair is influenced to some extent by the damage hole location, affecting load transfer and energy absorption. Coelho et al. [5] studied the performance of single-side and double-side patch repairs under impact loads using a central damage hole for experimental and simulation analysis, without a systematic study of location factors. Jefferson et al. [6] investigated the damage morphology of laminated structures and the performance of repaired structures under low-velocity impact loads, with both the impact and repair locations positioned at the center of the laminate for ease of measurement, but without indicating the effect of location on the repair and impact load. Overall, the composite repair field still pays limited attention to the critical parameter of “repair location” and lacks systematic conclusions that can be generalized to practical complex structures, requiring further study.

The cut-out depth, as one of the most critical geometric parameters in repair design, also has a fundamental impact on the performance of the repair structure. During the entire repair process, the damaged area must first be cleared, but the removal of the damaged portion must be performed on the basis of minimizing the weakening of the parent laminate, as the parent laminate is the primary load-bearing component. Insufficient removal may leave residual damage, while excessive removal may severely weaken the load-bearing capacity of the parent laminate and increase the burden on the patch and adhesive layer. Therefore, the reasonable cut-out depth is crucial to the repair performance. Santhanakrishnan et al. [7] used different treatment methods, including through-thickness and non-through-thickness simulations of the damaged area. The through-thickness approach refers to cases where the damage fully penetrates the thickness of the structure, whereas the non-through-thickness approach involves only partial thickness regions. The study indicates that the extent of damage removal is a key factor determining the overall repair performance. However, existing studies often couple the cut-out depth with other variables such as repair form and patch size, and lack independent, systematic, and refined parameter studies specifically focusing on the cut-out depth under the scenario of using scarf repairs and simulating underwater static loading conditions [8,9,10]. Therefore, establishing optimized recommended values for depth under specific working conditions is of great significance in improving the feasibility of engineering implementation. However, most existing studies are conducted under general mechanical loading conditions, and systematic parametric investigations of scarf-repaired composite structures under marine service environments (e.g., underwater hydrostatic pressure) remain relatively limited. In particular, in-depth analysis of the independent effect of cut-out depth, as a key parameter, is still lacking.

In summary, there are still gaps in current research: (1) Existing studies are largely based on the idealized assumption of a center-symmetric repair location, lacking systematic analysis of random damage locations in real structures, resulting in insufficient representativeness and engineering applicability; (2) The cut-out depth (i.e., the ratio of the damaged material removal depth to the parent laminate thickness) is a core design variable affecting repair effectiveness, but its impact mechanism on the mechanical performance of the repair structure has not been fully revealed. In particular, the transfer path of loads, stress redistribution patterns, and the intrinsic mechanism of coordinated working between the parent laminate, adhesive layer, and patch at different depths under load still lack systematic and in-depth research.

Based on these research gaps, this study employs a three-dimensional finite element simulation method, building upon a previously validated model, and utilizes a scarf repair configuration with a uniformly distributed pressure load simulating underwater service conditions. This loading condition is used to represent the underwater hydrostatic pressure scenario, making the analysis results more representative of the actual service environment of shipboard composite structures. By independently controlling and combining the two parameters of “repair location” and “cut-out depth”, multiple comparative scenarios are constructed, focusing on the study of key response characteristics such as the overall and local stress peaks, displacement field distribution, and adhesive layer stress state. The aim is to clarify how the repair location alters load distribution and transfer paths; secondly, to explore how the cut-out depth achieves a balance between material removal and load-bearing recovery. This paper first introduces the finite element model construction method, then analyzes and discusses the mechanical response patterns under the individual and combined effects of repair location and cut-out depth, and finally provides comprehensive suggestions and engineering application references. It should be noted that this study primarily focuses on the analysis of the influence mechanisms of key parameters (repair location and scarf depth) under the scarf repair configuration, and does not extend to other repair configurations (such as stepped-lap repair, double-patch repair, or hybrid repair structures). Different repair configurations may have their own applicability in practical engineering applications, and their mechanical response characteristics and parameter coupling relationships are more complex, which are beyond the scope of this study.

## 2. Methodology

**Figure 1:**
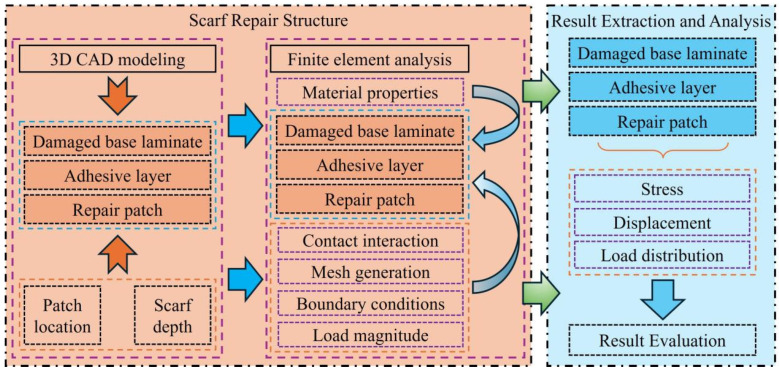
Overall technical roadmap.

This study adopts a three-dimensional finite element numerical simulation method to systematically analyze the impact of two key geometric parameters, repair location and cut-out depth, on the mechanical performance of composite repair structures. Unlike previous works that focused on comparing different repair forms, this study uses the scarf repair configuration as the sole research subject. The overall technical approach is shown in Figure 1, which includes four main stages: 3D modeling, finite element preprocessing, numerical calculation, and result extraction with mechanical response analysis. The specific steps are as follows:

(1) Construction of the 3D geometric model: Based on the pre-set repair location and cut-out depth parameters, a three-dimensional scarf patch repair geometric model is constructed, including the damaged parent laminate, adhesive layer, and patch. This model provides a unified and controlled geometric foundation for subsequent finite element analysis.
(2) Finite element model preprocessing: In Abaqus, material properties are assigned to the parent laminate, adhesive layer, and patch. The interface contact relationships or boundary conditions are properly set, and the mesh is generated according to the structural characteristics. Realistic boundary conditions and loads consistent with underwater service conditions are applied to ensure the physical validity of the simulation. A standardized and effective preprocessing process is a key step to ensure the reliability of the results.
(3) Numerical calculation and result extraction: After completing the model assembly and preprocessing, the calculation is submitted. The post-processing module is used to extract key response variables under different parameter conditions, including the overall and local maximum stress, magnitude of the displacement field, and stress concentration distribution at key locations such as the adhesive layer.
(4) Mechanical response analysis and parameter evaluation: By comparing the structural response characteristics under different repair locations and cut-out depths, the impact of each parameter on load-bearing capacity, deformation coordination, and potential failure risk is evaluated. Based on this, the differences and trends in mechanical response under various parameter combinations are identified, thereby providing useful reference and engineering guidance for the repair design of composite materials.

Building upon the four steps outlined above, a preliminary and clear technical approach has been established for this study. The following sections provide a detailed explanation of the model construction method, material parameter settings, mesh generation, and boundary load conditions, ensuring the reliability of the simulation analysis and the comparability of the results.

### 2.1. Scarf Patch Repair Structure Modeling

**Figure 2:**
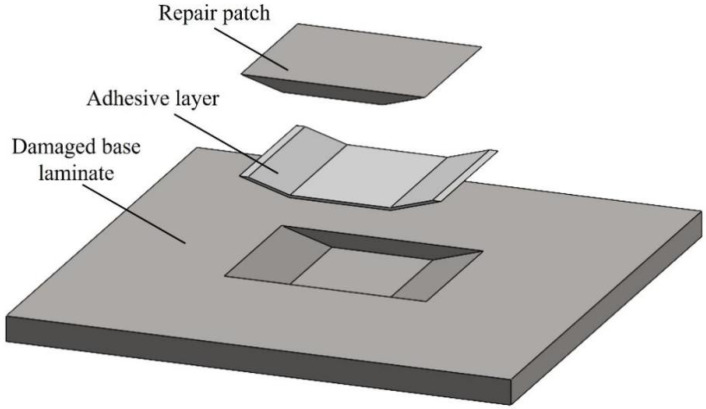
Schematic of scarf patch repair structure composition.

The scarf patch repair structure mainly consists of three parts: the parent laminate, adhesive layer, and patch [11,12], with the basic structure shown in Figure 2. During the modeling process, the scarf angle and repair configuration remain unchanged. The repair area is treated as a parametric analysis object, and different scenarios are constructed by adjusting the repair location and cut-out depth.

**Figure 3:**
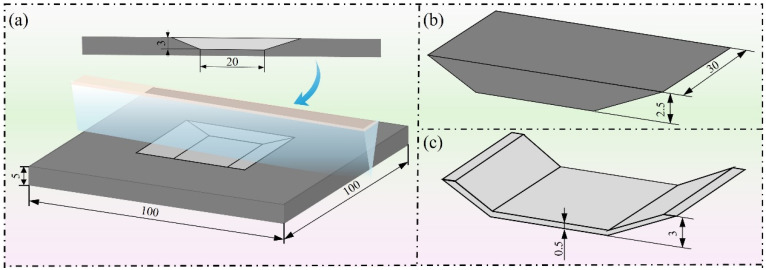
Schematic of repair structure modeling and layup design: (**a**) Parent laminate geometric dimensions; (**b**) Patch geometric dimensions; (**c**) Adhesive layer geometric dimensions.

The parent laminate is a square plate structure with dimensions set as length L = 100 mm, width W = 100 mm, and thickness H = 5 mm. A cut-out region is defined in a local area of the parent laminate to simulate the repair scenario after damage to the actual structure. The cut-out area removes a portion of material in the thickness direction, with a bottom length of 20 mm and width of 30 mm. The patch has a width of 30 mm and a thickness of 2.5 mm, ensuring geometric compatibility with the cut-out region, as shown in Figure 3a,b. It should be noted that a regular flat plate model with dimensions of 100 mm × 100 mm is adopted in this study, which represents an idealized modeling approach. The main purpose of this simplification is to establish a standardized parameter analysis framework, thereby reducing the interference of complex geometric factors (such as curved surfaces, stiffener distribution, and complex boundary conditions) on the analysis results, and thus more clearly revealing the influence mechanisms of repair location and scarf depth on the mechanical response of the repair structure.

### 2.2. Adhesive Layer Modeling Based on Cohesive Zone Model

The adhesive layer, as the key interfacial medium between the parent laminate and the patch, plays an important role in load transfer and interface stress distribution. To properly describe the mechanical behavior of the adhesive layer under external loading, the Cohesive Zone Model (CZM) is introduced in the adhesive layer region to model the adhesive-composite interface. In this study, the Cohesive Zone Model (CZM) is introduced to characterize the mechanical behavior of the adhesive interface between the patch and the parent structure, particularly the initiation and evolution of interfacial damage under loading. Compared with conventional continuum-based models, the CZM can effectively represent interfacial stiffness degradation and potential debonding behavior through a traction–separation relationship, making it more suitable for simulating interfacial responses in bonded structures.

The cohesive element uses a bilinear traction–separation law to describe the interface mechanical behavior [13]. Before damage occurs, the traction and separation displacement are linearly related, and its constitutive expression is given by:

(1) $$\left.\left\{\begin{array}{c} t_{n}=K_{nn}\delta_{n}\\ t_{s}=K_{ss}\delta_{s} \end{array}\right.\right.$$

Here, $t_{n}$, $t_{s}$, $\delta_{n}$, and $\delta_{s}$ represent the normal and tangential traction and separation displacements, respectively. $K_{nn}$ and $K_{ss}$ are the elastic stiffness coefficients of the cohesive elements along the two directions.

The initiation of damage at the adhesive interface is determined using the quadratic nominal stress criterion, expressed as:

(2) $${\left(\frac{t_{n}}{t_{n}^{0}}\right)}^{2}+{\left(\frac{t_{s}}{t_{s}^{0}}\right)}^{2}=1$$

where represents the Macaulay bracket, which returns the argument if positive and zero otherwise, used to prevent non-physical damage in the interface under purely compressive conditions. $t_{n}^{0}$ and $t_{s}^{0}$ represent the normal and tangential strengths of the interface, respectively. Once the damage initiation condition is satisfied, the interface enters the damage evolution stage, where the traction gradually degrades as the damage variable D increases [1,3]. The constitutive relationship of the cohesive element after damage can be expressed as:

(3) $$\left\{\begin{array}{c} t_{n}=(1-D)K_{nn}\delta_{n}\\ t_{s}=(1-D)K_{ss}\delta_{s}\;\;\;\; \end{array}\right.$$

Here, the damage variable D evolves monotonically from 0 to 1, corresponding to the undamaged and fully failed states of the interface, respectively.

Based on the aforementioned interface constitutive model, to enable the effective application of the cohesive zone model in numerical analysis, the adhesive layer is geometrically modeled as a solid body, with cohesive elements embedded in the adhesive region to describe the interface mechanical behavior. The adhesive layer thickness is set to 0.5 mm, with a coverage width of 30 mm. To ensure geometric compatibility with the parent laminate and patch, the overall height of the adhesive layer is set to 3 mm, as shown in Figure 3c. The adhesive material selected is Araldite^®^ AV138 epoxy resin [14,15,16], which is widely used due to its excellent mechanical properties and good interface compatibility with composite materials. The key material parameters of the adhesive layer are listed in Table 1, with an elastic modulus of 4890 MPa, shear modulus of 1560 MPa, tensile strength of 39.45 MPa, and shear strength of 30.2 MPa. Although the adhesive layer thickness may vary in practical applications, it is kept constant in this study in order to isolate the effects of the repair parameters. The adhesive thickness is kept constant to isolate the effect of repair parameters, although it may vary in practical applications. In practical engineering applications, the adhesive layer thickness should be optimized based on material properties and processing conditions.

By introducing representative cohesive parameters, the stress distribution characteristics and potential failure risks of the adhesive layer under different parameter conditions can be effectively characterized, providing a basis for evaluating the load-bearing capacity of the repair interface. It should be noted that this study primarily focuses on the initial damage behavior and stress distribution characteristics within the adhesive layer, and does not consider damage propagation or the structural response after complete failure, such as patch debonding or pull-off.

### 2.3. Material Modeling of Composite Parent Laminate and Patch

Both the parent laminate and the patch are made of carbon fiber reinforced polymer (CFRP) using T300 carbon fibers and epoxy resin. This material selection ensures material consistency and deformation compatibility between the repair area and the original structure. Both components use a symmetric orthogonal layup, with a layup sequence of [0/90]_6S_ [17,18]. Here, 0° and 90° denote the fiber orientations with respect to the reference direction. The notation [0/90]_6_ indicates that the sequence is repeated six times, and “s” denotes symmetry about the mid-plane. Therefore, the laminate consists of symmetrically arranged multiple 0°/90° plies. The parent laminate consists of 12 layers, and the patch consists of 6 layers.

The symmetric orthogonal layup structure helps to improve the tensile strength and bending stiffness of the laminate along the principal directions, enhancing the overall stability of the repair structure and reducing the risk of interlaminar failure. The main mechanical properties of the CFRP are shown in Table 2. The composite laminate is modeled as a homogenized orthotropic material using equivalent engineering constants. The longitudinal elastic modulus is 60 GPa, the transverse elastic moduli are 5 GPa, the in-plane shear modulus is 8.5 GPa, and the Poisson’s ratio is 0.13.

### 2.4. Mesh Generation and Boundary Condition Setup

**Figure 4:**
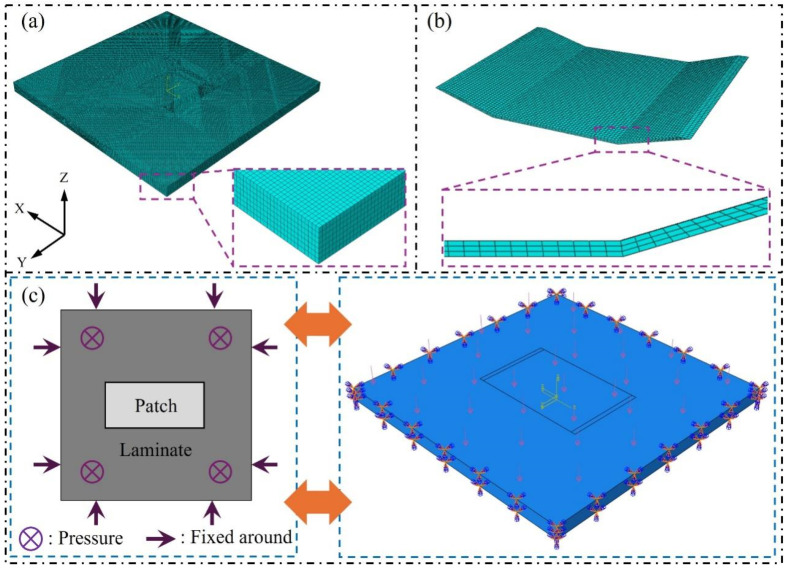
Schematic of finite element simulation analysis: (**a**) Mesh discretization of the parent laminate; (**b**) Mesh discretization through the thickness direction of the adhesive layer; (**c**) Boundary conditions and loading configuration.

In the finite element modeling process, to ensure the convergence and accuracy of the computational results, both the parent laminate and the patch are discretized using three-dimensional solid elements (C3D8R) [19,20]. The meshes are generated by sweeping. A global mesh size of 1 mm is used for the parent laminate region, with reasonable divisions along the ply direction, as shown in Figure 4a. Considering the large stress gradients in the patch and repair regions, a finer mesh size of 0.5 mm is applied to the patch region for higher resolution.

The adhesive layer region is also modeled using cohesive elements (element type: COH3D8), with the thickness direction divided into three layers to more accurately capture the interface shear stress distribution and potential damage evolution. The mesh division in the thickness direction of the adhesive layer is shown in Figure 4b.

A relatively refined mesh is adopted in this study, with local mesh refinement applied in the cut-out region where stress concentration occurs. It should be noted that a systematic mesh convergence study has not yet been conducted in the present work, which may affect the quantitative accuracy of local stress results. However, since the focus of this study is on comparative analysis and identifying relative trends among different parameter cases, this limitation does not affect the main conclusions.

To simulate the service conditions of composite repair structures in practical engineering applications, such as ship hulls, the outer surfaces of the parent laminate are fixed, restricting all six degrees of freedom to approximate the support conditions in real working environments, as shown in Figure 4c.

For the loading conditions, a uniform normal pressure is applied to the surface of the model to simulate the underwater hydrostatic pressure. The load value is set at 0.1 MPa, corresponding to the hydrostatic pressure level at a depth of approximately 10 m and is estimated based on the equation p=ρgh.

It should be noted that the loading condition adopted in this study represents an idealized static loading scenario, without considering complex marine effects such as wave impact and transient loads. Therefore, this loading does not represent extreme service conditions, but rather serves as a baseline case for parametric comparison.

By maintaining consistency in the load form and material parameters, the differences in the mechanical responses under different scenarios are solely caused by the repair location and cut-out depth, ensuring the reliability of the parameter analysis.

After the numerical computation is completed, key mechanical response indicators are extracted for different parameter conditions, including the maximum equivalent stress of the entire structure, maximum displacement response, and the stress concentration distribution in the adhesive layer region. By comparing the response differences under different repair locations and cut-out depths, the effects of parameter variations on structural load-bearing capacity, deformation coordination, and potential failure risks are systematically evaluated. This approach helps to identify the mechanical response characteristics and their variation trends under different parameter combinations, thereby providing useful reference and engineering guidance for parameter selection in scarf repair structures. It should be noted that, in order to systematically investigate the influence mechanisms of repair location and cut-out depth on the mechanical response of the structure, a regular plate model with idealized boundary conditions is adopted in this study. Within this modeling framework, factors such as curvature effects, stiffening structures, complex constraint conditions, and multi-axial coupled loading that may exist in actual shipboard composite structures are not considered. In addition, only static loading under hydrostatic pressure is considered, and the effects of wave impact, cyclic loading, and environmental factors (such as temperature and humidity) on the performance of the repaired structure are not addressed. Therefore, the results of this study are primarily intended to reveal the influence of different repair parameters on load transfer paths, stress distribution characteristics, and interfacial responses, rather than for direct quantitative prediction of real engineering structures. Future work will further incorporate curved structural models, complex service loading conditions, and environmental coupling effects to improve the engineering applicability of the analysis results.

## 3. Result and Discussion

### 3.1. Effect of Different Cut-Out Locations on Repair Structure Performance

In the composite material structure repair process, the repair location is one of the key factors influencing the repair effectiveness and the mechanical performance of the repaired structure. The choice of repair location not only determines the distribution characteristics of stress concentration and crack propagation but also directly affects the overall stability and long-term durability of the structure. Previous studies have shown significant differences in the maximum load-bearing capacity under low-velocity impact loads for different hole locations, indicating that the repair location has an impact on load absorption and energy dissipation capacity [3]. Therefore, systematically analyzing the effect of repair location variation on the mechanical response of the repair structure is crucial for optimizing repair schemes and improving engineering applicability.

**Figure 5:**
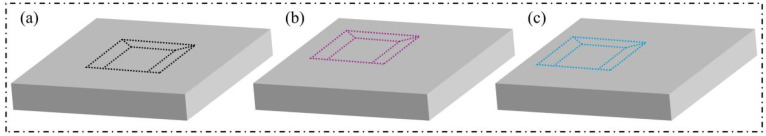
Schematic of three typical repair locations in composite repair area: (**a**) Center-symmetric repair; (**b**) Diagonal-Asymmetric repair; (**c**) Edge-Unidirectional repair.

Based on this understanding, this study selects three representative repair location schemes for comparative analysis, keeping the repair geometry and material parameters consistent. These schemes are the center-symmetric repair location (location a), diagonal-asymmetric repair location (location b), and edge-unidirectional repair location (location c), as shown in Figure 5. A uniform normal load of 0.1 MPa is applied to all scenarios to simulate the hydrostatic pressure environment at a depth of approximately 10 m, ensuring that the mechanical responses between different repair locations are comparable.

To quantitatively compare the influence of repair location on structural mechanical performance, the peak stress and maximum displacement of the entire structure, patch, and adhesive layer for the three repair configurations are summarized in Table 3.

It should be noted that to avoid the interference of geometric shape differences on the result analysis, this study uses the scarf patch repair structure as the research subject. By fixing the repair configuration and only changing the spatial position of the repair area in the parent laminate, the subsequent analysis can more clearly reveal the effect of repair location variation on load distribution, stress concentration, and structural deformation behavior.

**Figure 6:**
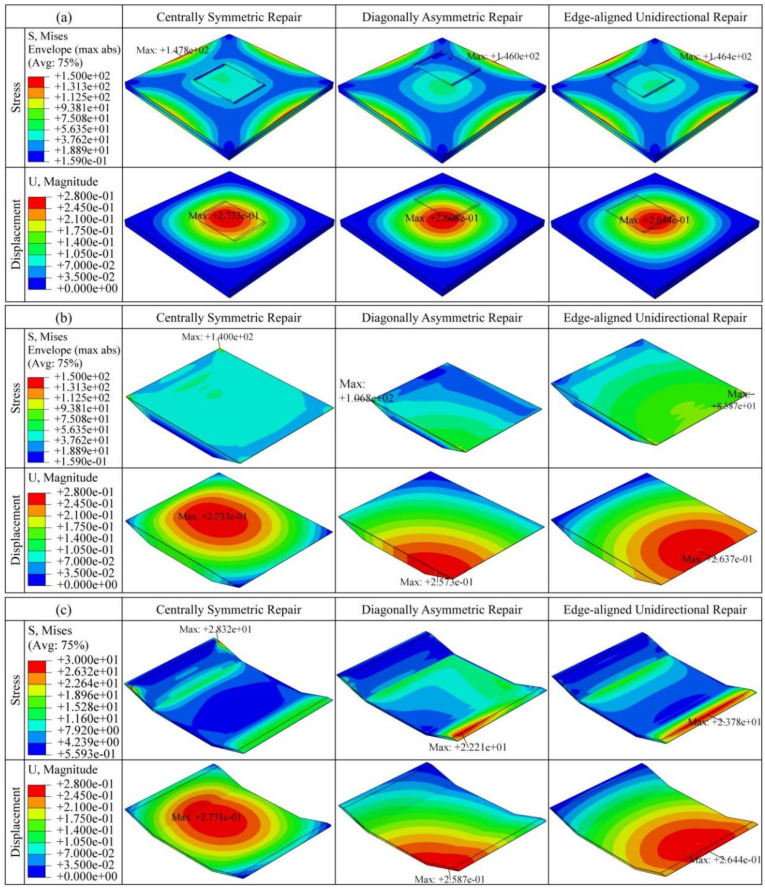
Stress and displacement distribution characteristics under three repair locations: center-symmetric repair, diagonal-asymmetric repair, and edge-unidirectional repair: (**a**) Overall distribution characteristics of the repair structure; (**b**) Local distribution characteristics of the patch area; (**c**) Local distribution characteristics of the adhesive layer area.

**Figure 7:**
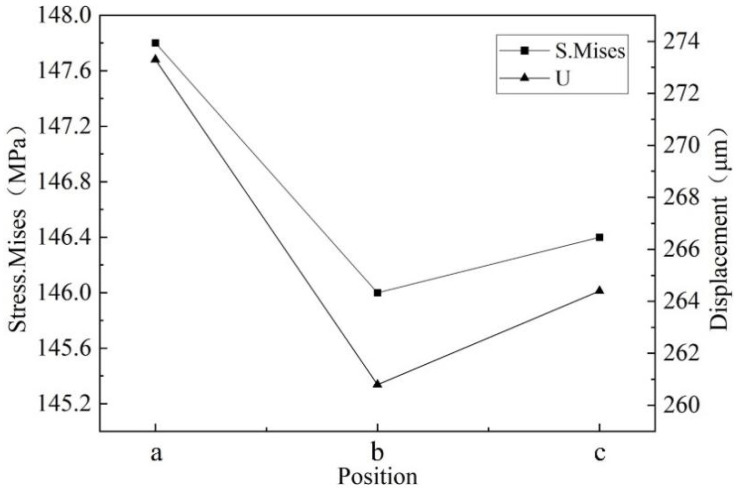
Stress and displacement response curves of the overall structure under different repair-location conditions.

Under the aforementioned conditions, numerical simulations are conducted for the three repair locations of the scarf patch repair structure. The simulation results are shown in Figure 6, which present the stress and displacement distribution characteristics of the entire repair structure, patch area, and adhesive layer area under different repair location conditions. Key response variables are further extracted to obtain the stress and displacement response curves for different repair locations, as shown in Figure 7. It should be emphasized that the stress analysis presented in this study is primarily based on relative comparisons among different repair scenarios. The results are intended to reveal the influence of repair location and cut-out depth on the structural response. Accordingly, the conclusions should be understood as trend observations rather than quantitative predictions of composite failure behavior.

The numerical results indicate that the repair location is a key factor affecting the mechanical response of the composite laminate. Based on the stress-displacement curves shown in Figure 7 and the numerical comparison presented in Table 1, the specific performance of the three repair locations is as follows:

From the perspective of stress distribution, it can be seen that the overall stress amplitude of the structure under different repair locations differs only slightly. Among the three repair configurations, the center-symmetric repair location (location a) has the highest maximum stress value, reaching 147.8 MPa. The overall stress amplitudes for the diagonal-asymmetric repair location (location b) and edge-unidirectional repair location (location c) are similar. In all three cases, the maximum stress is distributed along the structure’s boundary, indicating that no significant damage or failure has occurred within the repair area.

Further analysis of the stress distribution in the patch area reveals that the patch stress is highest at the center-symmetric repair location, with a peak value of 140 MPa. The stress levels at the diagonal-asymmetric repair location (location b) and edge-unidirectional repair location (location c) are relatively lower. This is mainly because, under the asymmetric repair condition, the carbon fiber composite parent laminate bears a larger proportion of the external load, thus reducing the stress levels in the patch and adhesive layer. The stress contour plots show that the stress distribution in the center-symmetric repair structure is relatively uniform, whereas locations b and c exhibit significant localized stress concentration phenomena. Although no damage occurs under the current loading conditions, such localized stress concentrations could become the main cause of initial damage or failure in the patch and adhesive layer under higher loading conditions.

In the stress analysis of the adhesive layer, it can be observed that the stress amplitude is highest at the center-symmetric repair location, lowest at the diagonal-asymmetric repair location (location b), and slightly higher at the edge-unidirectional repair location (location c) compared to location b. This indicates that in the center-symmetric repair structure, the adhesive layer bears more of the load transfer. Under the asymmetric repair conditions, some of the load is transferred by the central carbon fiber laminate, thus reducing the stress in the adhesive layer. Additionally, the maximum stress for locations b and c appears near the edge of the central region, exhibiting a stronger stress concentration trend. Under high-intensity or high-load conditions, these regions are likely to become potential sources of damage or fracture.

From the perspective of displacement distribution, the center-symmetric repair structure shows the largest overall displacement change, with the maximum displacement occurring at the patch location, reaching 273.3 μm. The maximum displacement in the adhesive layer is similar but slightly smaller. In contrast, the displacement amplitudes at the diagonal-asymmetric repair location (location b) and edge-unidirectional repair location (location c) are close, but the former is slightly smaller than the latter. Furthermore, the maximum displacement for the diagonal-asymmetric repair occurs in the parent laminate region, while for the edge-unidirectional repair, the maximum displacement is located in the adhesive layer.

In summary, the center-symmetric repair structure achieves a more uniform load distribution under the current loading conditions, creating good deformation coordination between the patch, adhesive layer, and parent laminate. This results in superior overall load-bearing capacity and structural stability. Given its regular structure, mature processing technology, and lower implementation costs, center-symmetric repair remains highly valuable in the practical repair of composite material structures.

### 3.2. Effect of Cut-Out Depth on Repair Structure Performance

In the composite patch repair process, the proper removal of the damaged area and the geometric design of the repair structure are key factors in restoring the structural mechanical performance. Among them, the cut-out depth, as an important parameter that directly represents the extent of material removal, not only affects the geometric characteristics of the repair area but also significantly influences the load transfer path, interface stress state, and the load-bearing and deformation behavior of the entire structure [12,21]. Building upon the previous analysis of repair location, this study further focuses on the effect of cut-out depth on the mechanical performance of the scarf patch repair structure.

**Figure 8:**
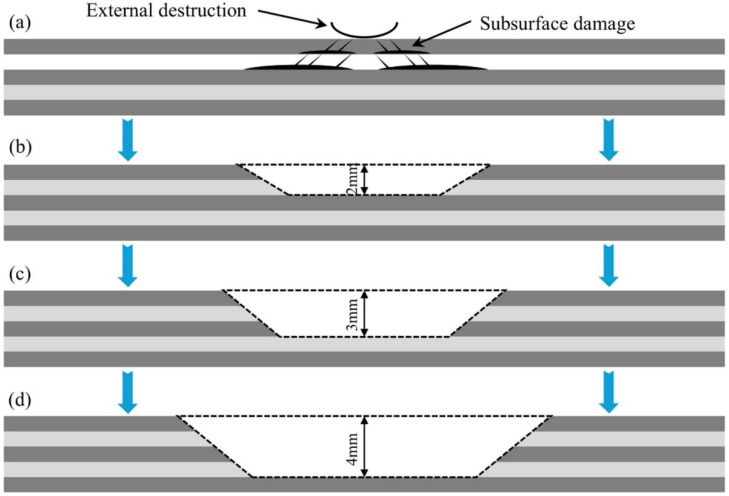
Schematic of composite repair with different cut-out depths: (**a**) Initial damage state; (**b**) Cut-out depth of 2 mm; (**c**) Cut-out depth of 3 mm; (**d**) Cut-out depth of 4 mm.

As shown in Figure 8, three representative cut-out depth conditions are selected for comparative analysis, namely 2 mm, 3 mm, and 4 mm, to simulate the repair state under different levels of damage removal. Under the condition of maintaining consistent repair configuration, material parameters, boundary conditions, and loading scenarios, three-dimensional finite element modeling and numerical analysis are performed for the parent laminate, adhesive layer, and patch at different cut-out depths. A uniform normal load of 0.1 MPa is applied to all scenarios to simulate the hydrostatic pressure at a depth of approximately 10 m.

**Figure 9:**
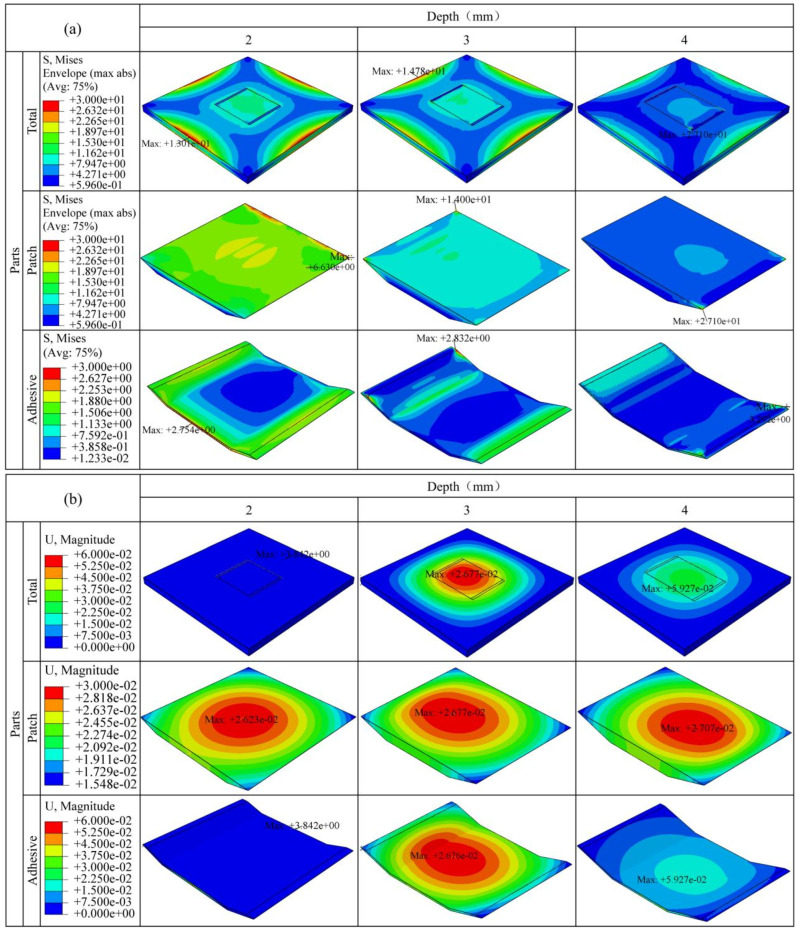
Stress and displacement distribution characteristics of the repair structure under different cut-out depths: (**a**) Stress distribution characteristics of the entire structure, patch, and adhesive layer; (**b**) Displacement variation distribution of the entire structure, patch, and adhesive layer.

Figure 9a shows the equivalent stress distribution characteristics of the entire repair structure, patch, and adhesive layer under different cut-out depth conditions. The simulation results demonstrate that the cut-out depth is a key factor influencing the stress response of the repair structure. As the cut-out depth increases, the peak stress shows an increasing trend: when the cut-out depth is 2 mm, the maximum equivalent stress of the entire structure is 13.0 MPa; when the cut-out depth increases to 3 mm, the peak stress slightly increases to 14.8 MPa, an increase of about 14%; further increasing the cut-out depth to 4 mm results in a significant increase in the peak stress to 27.1 MPa, which is approximately 83% higher than at the 3 mm depth. The high-stress areas are mainly concentrated within the repair area, especially at the corners of the patch, indicating that this region becomes more susceptible to stress concentration and failure at higher cut-out depths. Among them, this 83% increase in stress reflects a local relative trend; the absolute stress value remains far below the ultimate strength of the composite material. The core focus is on the migration of stress concentration regions and the changes in interfacial response.

To quantitatively evaluate the influence of cut-out depth on the mechanical performance of the repaired structure, the peak stress and maximum displacement of the entire structure, patch, and adhesive layer for the three cut-out depths (2 mm, 3 mm, and 4 mm) are summarized in Table 4.

From the numerical results presented in Table 4, it can be observed that, in terms of stress distribution morphology, under the 2 mm cut-out depth condition, a larger proportion of the stress is shared by the adhesive layer and the parent laminate, with the bottom region of the adhesive layer experiencing noticeable compressive stress, making it prone to local deformation under long-term service conditions. As the cut-out depth increases, the role of the patch in the overall load transfer gradually increases, and the stress becomes concentrated in the patch and interface regions, with a marked increase in peak stress. Meanwhile, the location of stress concentration in the adhesive layer shifts with the increase in cut-out depth: under the 2 mm condition, stress is mainly concentrated at the bottom edge of the adhesive layer; while under the 3 mm and 4 mm conditions, the stress is concentrated at the sharp corners of the adhesive layer, with the stress concentration being most significant under the 4 mm condition. These results indicate that as the cut-out depth increases, the stress distribution pattern shifts from being relatively dispersed to localized concentration, with the 3 mm cut-out depth showing a relative advantage in terms of stress uniformity.

Figure 9b shows the displacement response characteristics of the repair structure under different cut-out depth conditions. The findings demonstrate that the cut-out depth serves as a key design parameter governing the deformation behavior of the repair structure. When the cut-out depth is 2 mm, the structure experiences the maximum displacement under load, reaching 3.8 mm, which exceeds the allowable value, with significant damage and deformation in the adhesive layer. This suggests that the adhesive layer thickness is insufficient to effectively dissipate stress. Under the same loading conditions, when the cut-out depth increases to 3 mm, the maximum displacement decreases to 26.8 μm, primarily concentrated in the patch region. The displacement of the adhesive layer and patch is similar, with no significant damage occurring, indicating that a 3 mm cut-out depth can effectively improve the load transfer path and deformation coordination. When the cut-out depth increases to 4 mm, the maximum displacement rises to 59.3 μm, with the peak displacement occurring at the center of the adhesive layer bottom. The displacement variation in the patch is similar to the 3 mm case. This phenomenon suggests that when the cut-out depth is too large, the adhesive layer is more likely to undergo local bending under uniformly distributed load, causing the overall displacement to increase. Therefore, from the perspective of suppressing excessive deformation and preventing local failure of the adhesive layer, the 3 mm cut-out depth performs best in displacement control.

From a mechanical perspective, the superior performance at a 3 mm cut-out depth can be attributed to a favorable balance between stiffness matching and interfacial stress state. At this depth, the thickness ratio between the patch and the parent plate is approximately 0.5, allowing the patch to effectively participate in load transfer while maintaining sufficient residual stiffness in the parent structure, thereby avoiding excessive local bending deformation. Under this condition, the adhesive layer is primarily subjected to shear stress rather than peel stress, which helps delay damage initiation. In contrast, when the cut-out depth increases to 4 mm, the remaining thickness of the parent structure is reduced to only 1 mm, leading to a significant decrease in local bending stiffness. Under external pressure, the weakened parent structure is prone to local deflection, which introduces additional peel stresses at the adhesive interface. Meanwhile, the patch edges become regions of pronounced stress concentration, and the adhesive layer is located below the neutral axis, further exacerbating local bending effects and increasing the risk of interfacial debonding. When the cut-out depth is only 2 mm, the patch contributes insufficiently to load transfer, resulting in excessive stress being borne by the adhesive layer and a noticeable increase in overall displacement.

Comprehensive analysis of the stress distribution and displacement response indicates that the 3 mm cut-out depth performs optimally in composite material repair. Its stress distribution uniformity is better than that of the 2 mm and 4 mm cut-out depths, while also exhibiting the best displacement coordination, preventing adhesive layer damage or interface delamination risks. In contrast, the 2 mm depth lacks load-bearing capacity, leading to adhesive layer failure, while the 4 mm depth reduces repair reliability due to stress concentration and adhesive layer bending deformation. Therefore, the 3 mm cut-out depth has significant advantages in balancing stress distribution and displacement control and can be considered an optimal parameter for composite material repair.

## 4. Conclusions

This study addresses the issue of localized damage that may occur in shipboard composite structures during underwater service. Based on three-dimensional finite element numerical simulations, the mechanical response characteristics of scarf patch repair structures under different repair locations and cut-out depths were systematically investigated. Through comparative analysis of stress and displacement distribution in key regions of the entire structure, patch, and adhesive layer, the influence of repair parameters on repair performance was revealed. The main conclusions are as follows:

(1) The repair location has an important influence on the local stress distribution and interfacial response of the scarf repair structure. Different repair locations cause changes in the load transfer path and stress redistribution, affecting the stress level in the patch and adhesive layer as well as the degree of local stress concentration. The numerical results indicate that, although the effect on the global maximum stress is relatively limited, noticeable differences can be observed in the stress distribution near the patch edges and interfacial regions, which are critical for damage initiation. In addition, while the diagonal-asymmetric repair exhibits certain advantages in some mechanical response indicators, its structural asymmetry increases processing difficulty and complexity. In contrast, the center-symmetric repair maintains good mechanical performance while offering advantages such as a regular structural form, mature processing technology, and lower implementation costs, making it more feasible for practical engineering applications.
(2) Cut-out depth is a key geometric parameter that influences the load-bearing performance and interface reliability of the repair structure. Within the parameter range studied in this paper, when the cut-out depth is 3 mm, the repair structure performs optimally in terms of stress distribution uniformity and displacement coordination, effectively reducing stress concentration in the adhesive layer and thereby minimizing interface damage and delamination risks. In contrast, a smaller cut-out depth (2 mm) causes the adhesive layer to have insufficient load-bearing capacity, leading to potential interface failure; while a larger cut-out depth (4 mm) exacerbates local stress concentration and adhesive layer bending deformation, reducing the overall reliability of the repair structure. Within the limited parameter range and under the modeling assumptions considered in this study, the 3 mm cut-out depth performs best.

The optimal cut-out depth (3 mm) obtained in this study is based on a specific structural size (100 mm × 100 mm × 5 mm) and material system. In practical engineering applications, the optimal cut-out depth is generally closely related to the structural dimensions, laminate thickness, and mechanical properties of the composite and adhesive. Therefore, the present results are more suitable for revealing the trend of how cut-out depth influences the structural response, rather than providing directly applicable parameter values. Verification and optimization based on actual engineering conditions are recommended.

Potential interaction effects between repair location and cut-out depth may exist. Although these parameters are analyzed independently in this study, their combined effects may influence stress distribution and displacement behavior, particularly in asymmetric configurations or at larger cut-out depths. This aspect will be addressed in future work. Finally, it should be noted that the conclusions of this study are based on idealized models and parameter ranges, primarily reflecting the relative influence of different repair parameters on structural response. Their engineering applicability remains to be further validated under more complex structures and service conditions.

## Figures and Tables

**Table 1:** Epoxy resin Araldie^®^ AV138 materials properties.

Property	Symbol (Units)	Value
Density	*ρ* (kg/m^3^)	1250
Young’s modulus	E (MPa)	4890
Shear modulus	G (MPa)	1560
Damage initiation	T_1_ (MPa)	39.45
	T_2_ (MPa)	30.2
Fracture energy for damage evolution	$GI_{c}$ (N/mm)	0.20
	$GII_{c}$ (N/mm)	0.38

**Table 2:** CFRP Materials properties.

Property	Symbol (Units)	Value
Young’s modulus	E_1_ (GPa)	60
	E_2_, E_3_ (GPa)	5
Poisson’s ratio	ν_12_,ν_13_	0.13
	ν_23_	0.15
Shear modulus	G_12_,G_13_, G_23_ (GPa)	8.5
Density	*ρ* (kg/m^3^)	1450
Tensile Strength	Xt (MPa)	800
	Yt (MPa)	119
Compressive Strength	Xc (MPa)	350
	Yc (MPa)	84
Shear Strength	S_12_ (MPa)	45

**Table 3:** Peak stress and maximum displacement responses of the overall structure, patch, and adhesive layer under different repair locations.

Parts	Response Type	Centrally Symmetric Repair	Diagonally Asymmetric Repair	Edge-Aligned Unidirectional Repair
Total	Stress (MPa)	140.0	106.8	85.9
	Displacement (μm)	273	260	264
Patch	Stress (MPa)	140.0	106.8	85.9
	Displacement (μm)	273	257	264
Adhesive	Stress (MPa)	28.3	22.2	23.8
	Displacement (μm)	273	259	264

**Table 4:** Peak stress and maximum displacement responses of the overall structure, patch, and adhesive layer under different cut-out depths.

Response Type	Parts	Depth (2 mm)	Depth (3 mm)	Depth (4 mm)
Stress (MPa)	Total	13.0	14.8	27.1
	Patch	6.6	14.0	27.1
	Adhesive	2.8	2.8	3.2
Displacement (μm)	Total	3842.0	26.8	59.3
	Patch	26.2	26.8	27.0
	Adhesive	3842.0	26.8	59.3

## Data Availability

The raw data supporting the conclusions of this article will be made available by the authors on request.
